# CT-guided ^125^I brachytherapy on pulmonary metastases after resection of colorectal cancer: A report of six cases

**DOI:** 10.3892/ol.2014.2649

**Published:** 2014-10-30

**Authors:** SHUYUAN SHI, JINGKUI YANG, DAQIANG SUN

**Affiliations:** 1 Department of Thoracic Surgery, Second Hospital of Tianjin Medical University, Tianjin 300211, P.R. China; 2 Department of Thoracic Surgery, Tianjin Chest Hospital, Tianjin 300051, P.R. China

**Keywords:** colorectal cancer, pulmonary metastases, ^125^I seeds, computed tomography-guided

## Abstract

Colorectal cancer (CRC) is one of the most common malignancies in the world and distant metastasis is the main cause of cancer-related mortality. Percutaneous computed tomography (CT) guided radioactive ^125^I seed implantation (CTRISI) is a minimally invasive technique used to treat pulmonary metastases in CRC patients. In the present study, following colorectal cancer resection, six patients with pulmonary metastases were treated with computed tomography (CT)-guided percutaneous implantation of radioactive ^125^I seeds. At six months following seed implantation, CT examination was performed and compared with the images captured prior to the treatment. Of the total 13 lesions, four had disappeared, eight were reduced by >50% and one was enlarged, indicating that the local control rate was 92.3% (12/13). Overall, two patients developed intraoperative pneumothorax and one experienced hemoptysis subsequent to the procedure. Following a median follow-up period of 31 months, no local recurrence was observed in 12 of the metastatic lesions. The mean survival time was 32.7 months and the median survival time was 31 months.

## Introduction

Colorectal cancer (CRC) is the third most common malignancy in Western countries and is one of the leading causes of cancer-related mortality in China (1,2). In total, approximately 10–25% of patients with CRC develop pulmonary metastases (3). As no effective chemotherapy regimen has been developed for the treatment of pulmonary metastases of colorectal origin, surgery is the only potentially curative treatment option. However, only 2–4% of pulmonary metastases can be treated surgically and others require external beam radiotherapy and chemotherapy (4). Increasing the therapeutic doses of traditional external beam radiotherapy is challenging due to the severe side-effects. Although three-dimensional conformal radiation therapy (3D-CRT) and stereotactic external beam radiotherapy can administer tumoricidal doses, the side-effect of lung tissue damage remains a problem (5).

Percutaneous computed tomography (CT)-guided radioactive ^125^I seed implantation (CTRISI) is a minimally invasive modality. This brachytherapy is less time consuming and less traumatic, compared with the aforementioned treatments, and the side-effect of radiation damage is minimal (6). Patients are also more likely to accept this therapy due the minimally invasive nature of the technique. CTRISI has been used for the treatment of non-small cell lung cancer (NSCLC) (7,8). However, there have been few radioactive seed implantations for pulmonary metastases following resection of CRC and the efficiency of CTRISI has not been determined. The present study reports the preliminary results of six patients with pulmonary metastases following resection, who could not tolerate a surgical procedure and therefore, underwent CT-guided ^125^I brachytherapy.

## Materials and methods

In total, six patients, three males and three females, with an ages range of 68–86 years (mean ± standard deviation, 76.0±7.6 years), with pulmonary metastases following colon cancer resection, were treated with percutaneous CTRISI at the Department of Thoracic Surgery, Second Hospital of Tianjin Medical University (Tianjin, China) between November 2002 and May 2010. Informed consent was obtained from the subjects and the present study was approved by the Ethics Committee of Tianjin Medical University. The patient characteristics are shown in Table I. Of the total 13 metastatic lesions, eight were located in the left lung and five in the right lung. In total, 10 were located in the lung and three were located beneath the hilum of the lung. The average diameter was 2.8±1.5 cm (range, 1–6 cm) and the average volume was 29.5±29.4 cm^3^. A complication of right supraclavicular lymph node metastasis was observed in one case and subsequently received seed implantation.

The CT-guided brachytherapy procedure was carried out as previously described (9). Prior to the procedure, a treatment plan was prepared for each patient using a computerized treatment planning system (TPS; Prowess Panther, Prowess Inc., Concord, CA, USA) based on the CT images of the patients. The TPS generated a dose-volume histogram (DVH) and isodose curves of various percentages, and calculated the position (coordinates) of the brachytherapy applicator, dose and number of implanted seeds (Table II). Under local anesthesia, interstitial needles (Medical Device Technologies, Inc., Gainesville, FL, USA) were inserted into the tumor at ~0.5 cm intervals. Each ^125^I seed was implanted within an average of 1 cm^3^ of the tumor. The average planning target volume (PTV) of the 13 metastatic lesions was 32.1±30.1 cm^3^, and the average number of implanted seeds was 28±14.4 seeds. The average dose of the target area was 157.3±11.6 Gy, with a median dose of 152.4 Gy. The dose covered 90% of the volume (D_90_), 88.4±7.3 Gy, and the volume that received >90% of the prescribed dose (V_90_) was 31.5±29.5 cm^3^. Once the implant was completed, a CT scan was performed to verify the position and intensity of the ^125^I seeds according to TPS. The six-month post-procedural follow-up was complemented by CT examination. The follow-up was completed in May 2010 (Table III).

## Results

The brachytherapy catheters and ^125^I seeds were satisfactorily placed in all patients. Of the six patients, three developed pneumothorax during the procedure. These patients subsequently received chest-tube drainage as a curative treatment for the pneumothorax, and two to three days following this, the condition was resolved. Hemoptysis (~20 ml) was observed in one patient; this ceased two days following the oral administration of carbazochrome salicylate (5 mg three times a day, for three days).

**Figure 1:**
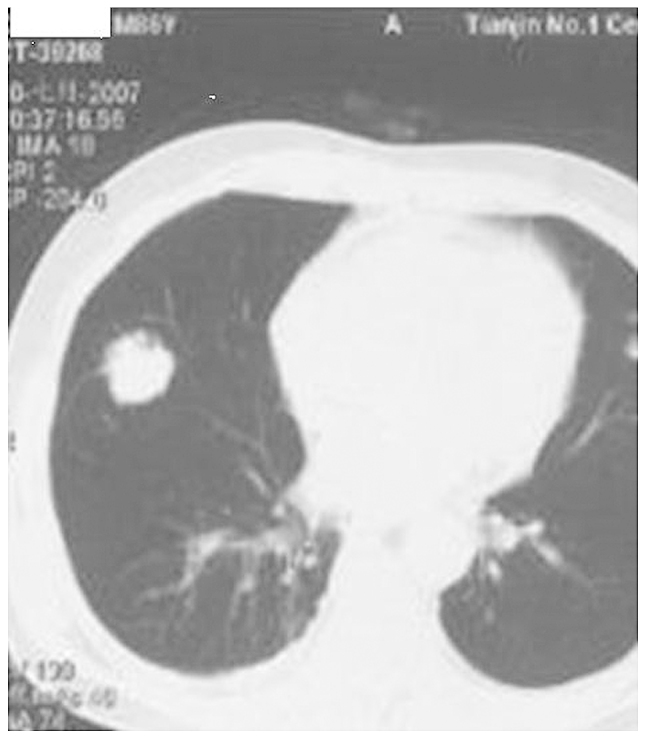
Male, 86 year-old patient 3 years following resection of colorectal cancer, revealing right lung metastases.

**Figure 2:**
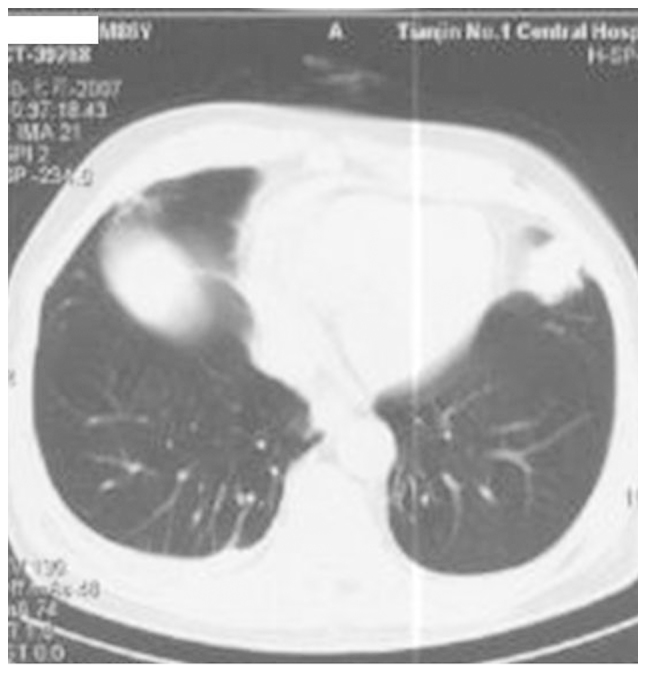
Male, 86 year-old patient 3 years following resection of colorectal cancer, revealing left lung metastases.

**Figure 3:**
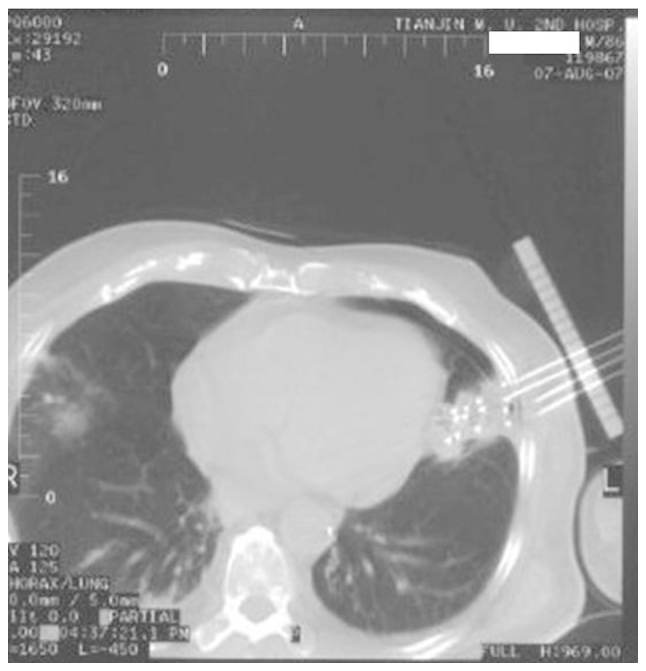
CT-guided ^125^I seed brachytherapy.

**Figure 4:**
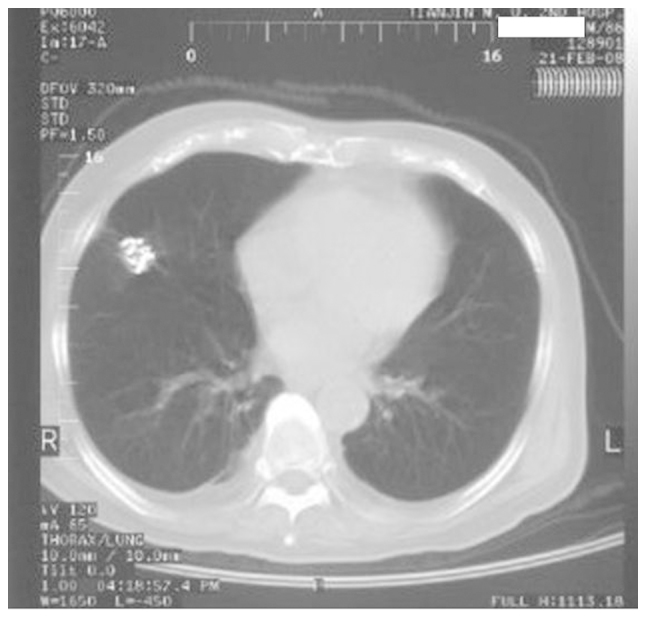
Right pulmonary metastases was reduced by >50% following ^125^I brachytherapy for six months.

**Figure 5:**
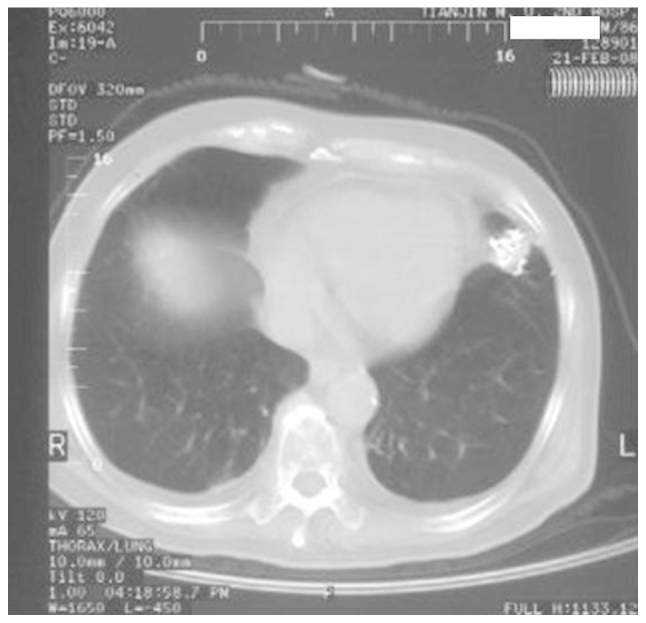
Left pulmonary metastases reduced by >50% following ^125^I brachytherapy for six months.

Compared with the CT images captured prior to the procedure, the CT images obtained at the six-month follow-up revealed that four masses had been completely removed by the treatment and eight masses had been reduced in size by >50%. The CT images of one 86-year-old male patient prior to and following the procedure are shown in Figs. 1–5. Overall, only one mass was enlarged, indicating that the local control rate was 92.3% (12/13). None of the patients developed radioactive pneumonia or reduction in peripheral-blood granulocytes. Following a median follow-up period of 31 months (32.7±16.6 months; range, 8–53 months), no local recurrence was observed for the 12 metastatic lesions. Of the two patients with poorly-differentiated adenocarcinoma, one suffered from pulmonary metastases, complicated by right supraclavicular lymph node metastasis six months following radical resection. One of these patients succumbed to the disease eight months following brachytherapy and the other succumbed 29 months following brachytherapy. The four patients with well-differentiated adenocarcinoma succumbed to the disease 49, 53, 33 and 24 months following brachytherapy. The mean survival time was 32.7 months and median survival time was 31 months.

## Discussion

The present study demonstrates that percutaneous CTRISI is a feasible and promising, minimally invasive modality for controlling the growth of pulmonary metastases following CRC resection, particularly in the 12 months following surgery. Although one patient experienced hemoptysis and three patients suffered pneumothorax, these side-effects could be controlled, indicating that CTRISI remains a safe treatment method in this patient population.

The target area may tolerate sustained, closer high-dose irradiation through the conformal implantation of seeds into the interior of the tumor, overcoming target volume motion, so that the local control rate can be elevated. Independent or separate entity radiotherapy studies have demonstrated that local control of the tumor body is likely to be markedly intensified following irradiation with a bioeffect dose of 90–100 Gy. Martínez-Monge *et al* used CT-guided permanent brachytherapy to treat seven patients with early-stage T1N0M0 NSCLC. The median dose was 144 Gy. This study found that one patient developed a focal pneumonitis three months following the treatment, and no patients developed local or regional failure within a 13 month follow-up period (7). In the present study, the mean tolerance dose of PTV (gross tumor volume + 0.5 cm) was 157.3 Gy, with a median dose of 152.4 Gy. The lesions were irradiated with a dose of approximately twice the PD and a local control rate of 92.3% was achieved, which is similar to the effective rate of 93.8% (10) for seed implantation for pulmonary metastases and 87% (11) for 3D conformal external beam radiotherapy, as previously reported in the literature, demonstrating good therapeutic effects. This may be associated with the high-dose irradiation of the small target area as well as with the sensitivity of well-differentiated adenocarcinoma to γ-ray radiation.

The seeds can be accurately and evenly implanted into the target area under CT guidance. Patients with the target area located at the tumor center should undergo stereotactic puncture following contrast-enhanced CT scanning of blood vessels. The puncture needlepoint can be close to the heart and great vessels without injury to them. In the present study, D_90_ 88.4 Gy (>PD) revealed a uniform and reasonable dose distribution.

The ^125^I-ray seed ray decays in an exponential manner with distance, which can reduce damage to tissues around the target area. The occurrence rate of radioactive pneumonia has been reported to be 44% (12) when the average therapeutic dose of the 3D conformal radiotherapy is 60 Gy. In the present study, no radioactive pneumonia was observed at PD 80 Gy, suggesting that seed implantation causes less damage as a result of radioactivity than conventional radiotherapy. Puncture-induced pneumothorax and hemoptysis can be observed on CT imaging and can be treated using conventional methods.

In conclusion, CTRISI is a safe and minimally invasive treatment modality for metastases from CRC that may aid in prolonging the survival rate in patients who cannot undergo pulmonary resection for metastases. While these results are promising, future studies including an increased number of cases, are required to gain further information with regard to CTRISI for the treatment of pulmonary metastases following CRC resection.

## Figures and Tables

**Table I:** Patient characteristics.

Patient	Gender	Age, years	Time to recurrence, months	Adenocarcinoma pathology	Number of lesions	Location	Type	Diameter, cm	Volume, cm^3^	Reason for seed implantation
1	Female	71	4	Poorly-differentiated	1	Adjacent to aortic arch	Central	4	60	Beside the aortic arch
2	Female	71	12	Well-differentiated	1	Lower right lung	Peripheral	4	48	Economic reasons
3	Male	68	12	Well-differentiated	2	Upper right lung	Peripheral	2	10	Multiple pulmonary metastases
						Upper left lung	Peripheral	2	15	
4	Female	75	6	Poorly-differentiated	1	Right pulmonary hilum	Central	5	80	Lymph node metastasis
5	Male	85	36	Well-differentiated	2	Right pulmonary hilum	Central	6	96	Multiple pulmonary metastases
						Upper right lung	Peripheral	2	10	
					2	Upper left lung	Peripheral	2	15	
						Upper left lung	Peripheral	3	27	
					1	Lower left lung	Peripheral	3	25	
6	Male	86	45	Well-differentiated	3	Upper left lung	Peripheral	1	2	Multiple pulmonary metastases
						Upper left lung	Peripheral	1	2	
						Upper left lung	Peripheral	2	8	

**Table II:** Seed implant characteristics.

Patient	Implantation time (yyyy/mm)	Location	PTV, cm^3^	Planned number	Number implanted	Average dose, cGy	D_90_, cGy	V_90,_ cm^3^	Complication
1	2002.11	Upper left lung beside aortic arch	64.6	35	35	14921.4	8880	63.3	Pneumothorax
2	2004.3	Lower right lung	53.1	34	35	16635.1	9200	50.8	
3	2005.3	Upper right lung	11.9	12	15	16923.3	9760	11.8	
		Upper left lung	17.1	15	20	16390.9	9280	16.7	
4	2005.7	Right pulmonary hilum	76.9	43	50	17659.8	9920	76.1	Hemoptysis
5	2007.8	Right pulmonary hilum	93.1	43	50	16532.1	8960	90.4	Pneumothorax
		Upper right lung	10.8	8	10	15238.4	8206	10.3	
		Upper left lung	16.8	13	13	16253.9	8240	16.1	
		Upper left lung	32.1	28	27	15231.7	8170	31.8	
		Lower left lung	30.9	25	25	15085.5	7920	30.2	
6	2008.5	Upper left lung	1.7	4	4	13822.4	8320	1.6	
		Upper left lung	1.7	4	4	13822.4	8320	1.6	
		Upper left lung	8.4	10	10	16003.0	9920	8.4	

PTV, planning target volume; D_90_, dose required to cover 90% of the volume; V_90_, volume that received >90% of the dose.

**Table III:** Follow-up and outcome of the disease.

Patient	Number of lesions	Location	Lesion status at six-month follow-up	Date of death (month/year)	Survival time, months
1	1	Adjacent to aortic arch	Decreased by 50%	04/2005	29
2	1	Lower right lung	Decreased by 50%	04/2008	49
3	2	Upper right lung	Disappeared	08/2009	53
		Upper left lung	Disappeared		
4	1	Right pulmonary hilum	Enlarged	03/2006	8
5	2	Right pulmonary hilum	Decreased by 50%	05/2010	33
		Upper right lung	Decreased by 50%		
	2	Upper left lung	Decreased by 50%		33
		Upper left lung	Decreased by 50%		
	1	Lower left lung	Decreased by 50%		
6	3	Upper left lung	Disappeared	05/2010	24
		Upper left lung	Disappeared		
		Upper left lung	Decreased by 50%		
